# The membrane: transertion as an organizing principle in membrane heterogeneity

**DOI:** 10.3389/fmicb.2015.00572

**Published:** 2015-06-12

**Authors:** Kouji Matsumoto, Hiroshi Hara, Itzhak Fishov, Eugenia Mileykovskaya, Vic Norris

**Affiliations:** ^1^Department of Biochemistry and Molecular Biology, Graduate School of Science and Engineering, Saitama University, SaitamaJapan; ^2^Department of Life Sciences, Ben-Gurion University of the Negev, Beer-ShevaIsrael; ^3^Department of Biochemistry and Molecular Biology, University of Texas Medical School at HoustonHouston, TX, USA; ^4^Laboratory of Microbiology Signals and Microenvironment EA 4312, Department of Science, University of Rouen, Mont-Saint-AignanFrance

**Keywords:** membrane, transertion, hyperstructures, lipid domain, *Escherichia coli*, Bacillus subtilis

## Abstract

The bacterial membrane exhibits a significantly heterogeneous distribution of lipids and proteins. This heterogeneity results mainly from lipid–lipid, protein–protein, and lipid–protein associations which are orchestrated by the coupled transcription, translation and insertion of nascent proteins into and through membrane (transertion). Transertion is central not only to the individual assembly and disassembly of large physically linked groups of macromolecules (*alias* hyperstructures) but also to the interactions between these hyperstructures. We review here these interactions in the context of the processes in *Bacillus subtilis* and *Escherichia coli* of nutrient sensing, membrane synthesis, cytoskeletal dynamics, DNA replication, chromosome segregation, and cell division.

## Introduction

The laterally heterogeneous distribution of the lipid and the protein components of bacterial cell membranes is the current paradigm, having replaced the homogeneous distribution of these components that is assumed in the fluid mosaic membrane model. This heterogeneity is involved in producing the specific environments needed for membrane proteins to participate in the many important processes that are associated with cell membranes. This raises the question of the nature of the mechanisms responsible for membrane heterogeneity.

**FIGURE 1:**
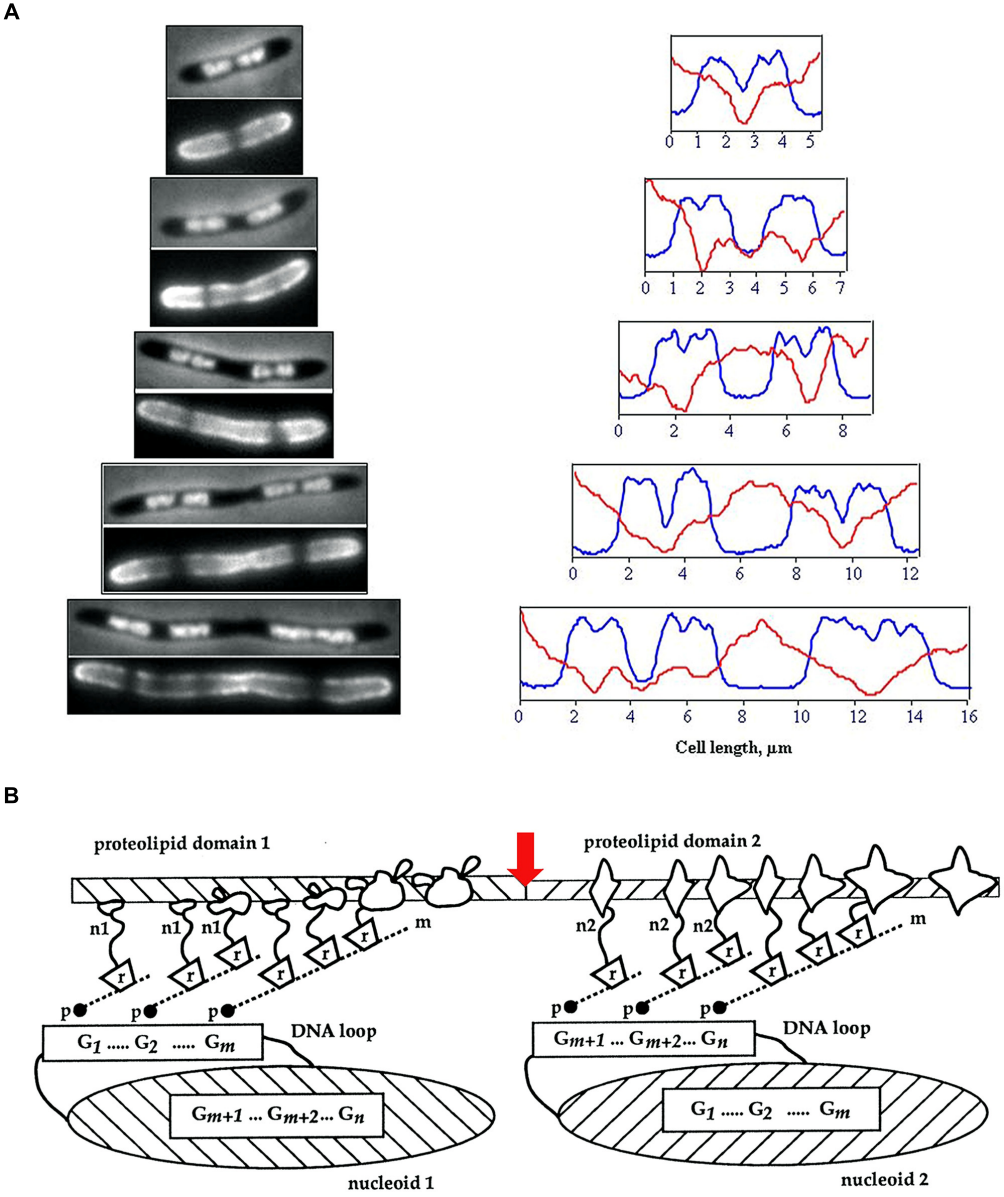
**(A)** Visualization of membrane heterogeneity in *Escherichia coli* cells by fluorescent microscopy. Left: image of an *E. coli* cell division mutant *pbpB*(ts) transferred to the non-permissive temperature (time zero, bacterium at the top), double-stained with DAPI (top half of image) and FM4-64 (bottom half of image) for visualization of nucleoids and membrane, respectively; adapted from Fishov and Woldringh (1999), © John Wiley and Sons. On the right, plots of fluorescence intensity profiles along filaments (DAPI, blue line; FM4-64, red). **(B)** The relationship between proteolipid domains, transertion, and clusters of genes either “looped out” from or buried within the nucleoid. n1, n2, nascent proteins; m, mRNA; r, ribosomes; p, RNA polymerase; G, gene clusters; red arrow indicates division site; adapted from Norris and Madsen (1995), © Elsevier.

One of the main causes of large-scale heterogeneity in bacterial membranes is the coupled transcription, translation and insertion of nascent proteins into and through membrane, *alias* transertion (Norris and Madsen, 1995; Binenbaum et al., 1999; Bakshi et al., 2014; **Figure 1**). The tethering of nascent proteins orchestrates interactions that result in the formation of membrane domains. These interactions include protein–protein interactions and lipid–protein interactions – and therefore also include lipid–lipid interactions (Vereb et al., 2003). The multitude of processes in which transertion is implicated has led to it being proposed as a powerful force in maintaining the nucleoid in expanded state, in the integrative sensing of metabolic activity and in coupling chromosome segregation to cell division (Norris and Amar, 2012; Fishov and Norris, 2012b).

It has long been evident that bacteria are highly structured (for references see Norris et al., 1994). It is now emerging that this structuring is in the form of *hyperstructures* which are large, physically linked, assemblies of macromolecules that serve specific functions (Norris et al., 2007a). Transertion plays a central role in the formation of one class of hyperstructures and also in the dialog between these hyperstructures that determines the behavior of the cell itself. Here, we review membrane heterogeneity focussing on transertion hyperstructures and their relationships with the processes of nutrient sensing, membrane synthesis, cytoskeletal dynamics, DNA replication, chromosome segregation, and cell division.

## Lipid Domains

### Cardiolipin and Other Anionic Phospholipid Domains

Although the lipids present in the inner membrane of *Escherichia coli* will spontaneously separate into domains *in vitro* (Zerrouk et al., 2008), until fairly recently, the lipid molecules in the membranes of bacterial cells were assumed to be homogeneously distributed, since the fluidity of biological membranes had become generally accepted following the publication of the fluid mosaic model (Singer and Nicolson, 1972). However, cell membranes must be laterally polarized to produce specific environments for certain membrane proteins, in particular, chemoreceptor proteins and proteins that promote polymerization of actin in eukaryotic hosts, as well as proteins involved in cell division at mid-cell and at asymmetric positions (Shapiro et al., 2002). Microscopic visualization of membrane lipids in cells has reinforced the view that bacterial membranes do possess structural heterogeneity. Uneven distribution of fluorescent lipophilic dyes and selective staining of septal regions has been observed in mycobacteria and an uneven distribution of fluorescence has been observed in lipophilic dye (FM4-64)-stained *E. coli* cells (Christensen et al., 1999; Fishov and Woldringh, 1999; **Figure 1**).

**FIGURE 2:**
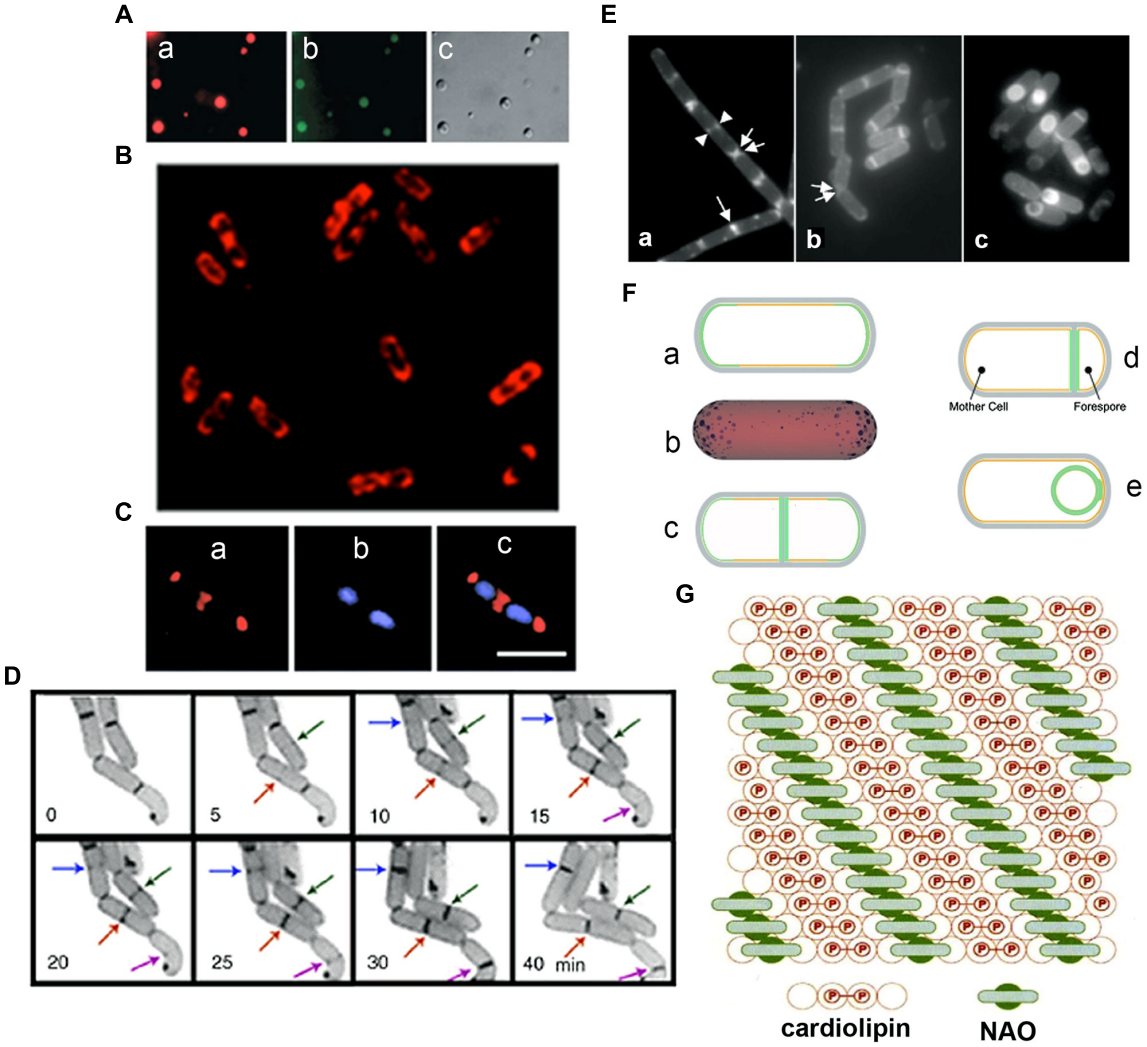
**Anionic phospholipid domains in *E. coli* and *B. subtilis* cells revealed by NAO staining**. **(A)** Fluorescence microscopy of cardiolipin (CL)-containing liposomes stained with NAO. Liposomes composed of CL and phosphatidylcholine (60 mol%/40 mol%). Excitation: 490 nm, emission: 617 nm (a) and 528 (b), DIC (c); modified from Mileykovskaya (2007), © John Wiley and Sons. **(B)** Deconvolved images of an optical section of *E. coli* W3899 stained with NAO. Cells were grown in LB media in the presence of 200 nM NAO to OD_600_ 1.5, and then immobilized on a microscope slide cover glass with poly-L-lysine; modified from Mileykovskaya and Dowhan (2009), © Elsevier. Excitation is 490 nm and emission is 617 nm. **(C)** *E. coli* cells stained with NAO (red) for CL (a) and DAPI (blue) for the nucleoid (b). Overlay of images (c) demonstrates localization of CL at the cell poles and at the division site; scale bar, 2.5 μM; modified from Mileykovskaya and Dowhan (2000), © American Society for Microbiology. **(D)** The dynamics of CL domain formation during the cell cycle of growing *E. coli*. Arrows of the same color indicate the progression of the corresponding NAO-stained structures with time; adapted from Mileykovskaya and Dowhan (2009). **(E)** Staining of wild type *B. subtilis* cells with NAO. Wild type *B. subtilis* 168 cells were harvested in exponential growth and sporulation phase (at T2 and T4) and stained with 100 nM NAO for 20 min. Fluorescence images of exponential growth (a) and sporulation phase cells at T2 (b) and T4 (c). An arrow indicates a sharp fluorescence band in the center of the cell. Two fluorescence dot structures in the cell center are indicated by a pair of arrowheads. Regions of NAO stained nascent poles in the cells that are separating are indicated with a pair of arrows. Panels are adapted from Kawai et al. (2004), © American Society for Microbiology. **(F)** Osmotic pressure and turgor-induced localization of lipid clusters during cell division and sporulation. During exponential growth, high-intrinsic-curvature clusters of CL localize to the poles of the inner leaflet [green curve (a) or blue clusters in (b)], driven by differences in membrane curvature. A lower osmotic-pressure differential across the septal/forespore-engulfing membrane [green area, (c)/(d), respectively] induces relocalization of the lipid clusters to the septal membrane; (d) CL clusters migrate along the continuous leaflet consisting of the inner leaflet of the mother cell and the outer leaflet of the forespore-engulfing membrane to localize around the spore due to low osmotic-pressure differential (green circle); modified from Huang et al. (2006) and Huang and Ramamurthi (2010), © John Wiley and Sons with permission. **(G)** The proposed arrangement of CL in the presence of NAO. A top view of the bilayer in which the hexagonal array of large circles represents the fatty acid chains is shown. The small internal circles containing P represent the phosphate groups, hydrogen-bonded tightly by the hydroxyl of the connecting glycerol, above the two central circles of the four fatty acid chains of CL (red). This tight array provides room for the NAO molecules (green) to stack in between the rows of CL head groups; adapted from Mileykovskaya et al. (2001), © Elsevier.

Unequivocal visualization of cardiolipin (CL) domains in the septal region and in the poles in the membranes of *E. coli* cells has been accomplished by means of the CL-specific fluorescent dye 10-*N*-nonyl-3,6-bis(dimethylamino)acridine (10-*N*-nonyl-acridine orange: NAO; Mileykovskaya and Dowhan, 2000, 2009; Mileykovskaya, 2007; **Figures 2A–C**). Time-lapse microscopy of *E. coli* cells showed positioning of NAO stained domains at nascent division sites and their gradual development into septal domains (Mileykovskaya and Dowhan, 2009; Mileykovskaya and Margolin, 2012; **Figure 2D**). NAO only binds to anionic phospholipids owing to an interaction between its quaternary amine and the phosphate residue of phospholipids and an intercalation of the hydrophobic acridine moiety into the membrane bilayer (Petit et al., 1992). The stoichiometry between NAO and monoacidic phospholipids is 1:1 and the emitted fluorescence is green whilst with CL, which contains two phosphate groups per molecule, NAO forms a dimer and the emitted fluorescence of the CL complex shifts to red due to the metachromatic effect of acridine molecules (Petit et al., 1994; Mileykovskaya et al., 2001; **Figure 2G**). CL domains have also been observed in *Bacillus subtilis* cells, both during exponential growth in the septal region and the poles and during sporulation in the engulfment and the forespore membranes (Kawai et al., 2004, 2006; **Figures 2E,F**). Note that in *B. subtilis* cells PE is also localized in polar and septal membranes (Nishibori et al., 2005). The CL localization to the polar membranes in *E. coli* is consistent with the increase in the content of CL in the membranes of minicells, which are rich in polar membranes (Koppelman et al., 2001).

An *E. coli*Δ*pgsA* mutant lacks phosphatidylglycerophosphate synthase, which catalyzes the committed step of biosynthesis of the major acidic phospholipids, and thus lacks phosphatidylglycerol (PG) and CL (both < 0.01% of total phospholipids); this mutant, which is viable if it also has an *lpp* mutation, accumulates the anionic biosynthetic precursors, phosphatidic acid (PA) and CDP-diacylglycerol (4.0 and 3.2%, respectively; Kikuchi et al., 2000; Shiba et al., 2004). PG is required for modification of prolipoprotein (the precursor of Braun’s lipoprotein, the product of *lpp*); cells lacking PG in an *lpp^+^* background are not viable because the accumulation of unmodified prolipoprotein in the inner membrane causes a tight membrane fusion (Suzuki et al., 2002). The viability of a Δ*pgsA lpp^-^* mutant suggests that the anionic phospholipids can substitute for one another in essential biological functions that include (1) the initiation of DNA replication, which depends on the rejuvenation of the DnaA protein by CL, and (2) the selection of the division site, which depends on the inhibition of inappropriate FtsZ polymerization in the polar regions by a complex, MinCD, with a high affinity for anionic phospholipids (Matsumoto, 2001; Szeto et al., 2002; Mileykovskaya et al., 2003; Norris et al., 2004; Mileykovskaya and Dowhan, 2005; Mazor et al., 2008; Vecchiarelli et al., 2014). In the Δ*pgsA* mutant cells, *N*-acyl-phosphatidylethanolamine (*N*-acyl-PE) and PA have been localized to polar and septal membrane domains (Mileykovskaya et al., 2009), indicating that these normally minor anionic phospholipids are segregated, like CL, into similar anionic lipid domains. Thus, *E. coli* has a mechanism for preferential segregation of anionic phospholipids to the polar and septal regions. One possible segregation mechanism depends on the shape of lipid molecules. ‘Cone-shaped’ lipid molecules, in which the cross-sectional area of the polar head group is less than the cross-sectional area of the hydrophobic domain, prefer regions of membranes that have a negative curvature; hence CL and PA are concentrated in negatively curved regions of the inner leaflet of the bacterial membranes (Cullis et al., 1983; Gennis, 1989; Seddon, 1990; Renner and Weibel, 2011; Jouhet, 2013; Renner et al., 2013). Using a mathematical model, this sensing of membrane curvature – and consequent positioning in the poles – has been attributed to stable, conical clusters of CL molecules forming due to the membrane being pinned to the cell wall, which is itself the result of the balance between the osmotic-pressure difference across the membrane and the inward pressure of the cell wall (Huang et al., 2006; Huang and Ramamurthi, 2010; **Figure 2F**).

A recent report from Weibel’s lab has claimed that there is no clear preference of NAO for binding to CL compared to PG and other anionic phospholipids, and that NAO produces an intense red-shifted fluorescence emission with PG, PA, and phosphatidylserine (PS) that is comparable to that with CL (Oliver et al., 2014). They suggested that not only CL but also PG is concentrated in the polar regions of *E. coli* cell membranes. However, they used much higher concentrations of NAO than previous authors used for bacterial staining (Mileykovskaya and Dowhan, 2000; Kawai et al., 2004; Romantsov et al., 2007, 2008; Rosch et al., 2007; Lobasso et al., 2009, 2013; Maloney et al., 2011; Muchová et al., 2011; Kicia et al., 2012; Tran et al., 2013). Moreover, PG microdomains, in contrast to CL domains, cannot sense membrane curvature, since the cross-sectional area of PG molecule is cylindrical and lamellar structures are preferred (Cullis et al., 1983; Gennis, 1989; Seddon, 1990). As Weibel’s lab had already demonstrated that the location of NAO-stained domains depends directly on membrane curvature (Renner and Weibel, 2011; Renner et al., 2013), this group did not propose a specific physical mechanism responsible for localizing anionic phospholipid domains stained by NAO (Oliver et al., 2014). In *B. subtilis*, phospholipid-specific dyes including FM4-64 are localized in a helical pattern extending along the long axis (Barák et al., 2008). Helical FM4-64 domains are missing in cells depleted of MurG, an enzyme involved in peptidoglycan synthesis, indicating a link between the helical domains and peptidoglycan synthesis (Muchová et al., 2011). As the helical structures are absent from the cells with repressed *pgsA* expression (in which the levels of PG and CL are much reduced; Hashimoto et al., 2009), the dyes are considered to be associated with the anionic phospholipids (Barák et al., 2008). As the CL-specific NAO dye is located in the septal regions and the poles of *B. subtilis* cells (Kawai et al., 2004), the principal component of the FM4-64 helical structures is likely to be PG (Barák et al., 2008). It has, however, been suggested that experiments showing changes in FM 4-64 staining of *B. subtilis* cells with repressed *pgsA* expression cannot serve as unambiguous evidence of FM 4-64 specificity for PG since, first, changes in the distribution of FM 4-64 resulted from the dissipation of membrane potential by the uncoupler CCCP and, second, the membrane potential was much lower in *B. subtilis* cells with repressed *pgsA* expression (Strahl et al., 2014). In *E. coli* no discernible helical structures were observed with FM4-64 staining (Barák et al., 2008) as reported previously (Fishov and Woldringh, 1999).

Microdomains that are functionally similar to the lipid rafts of eukaryotic cells have recently been found in *B. subtilis* membranes (Lopez and Kolter, 2010). These microdomains contain homologues of eukaryotic flotillins, YuaG and YqfA, referred to as FloT and FloA, respectively (see Heterogeneous Distribution of Envelope and Envelope-Associated Proteins). The distribution of these microdomains was in a punctuated pattern along the cytoplasmic membrane (Donovan and Bramkamp, 2009; Lopez and Kolter, 2010), which differs from the patterns of FM4-64-stained helices and of CL-domains in the septal region and the poles. YisP is involved in biofilm formation in *B. subtilis* and has been predicted to produce C30 isoprenoids; the enzyme acts as a phosphatase, catalyzing formation of farnesol from farnesyl diphosphate (Feng et al., 2014). Inhibition of formation of these microdomains using zaragozic acid impaired biofilm formation and protein secretion but not cell viability and it has been suggested that polyisoprenoids are constituents of the microdomains (Lopez and Kolter, 2010; Feng et al., 2014; Bramkamp and Lopez, 2015).

### Lipid Synthases and Membrane Domains

**FIGURE 3:**
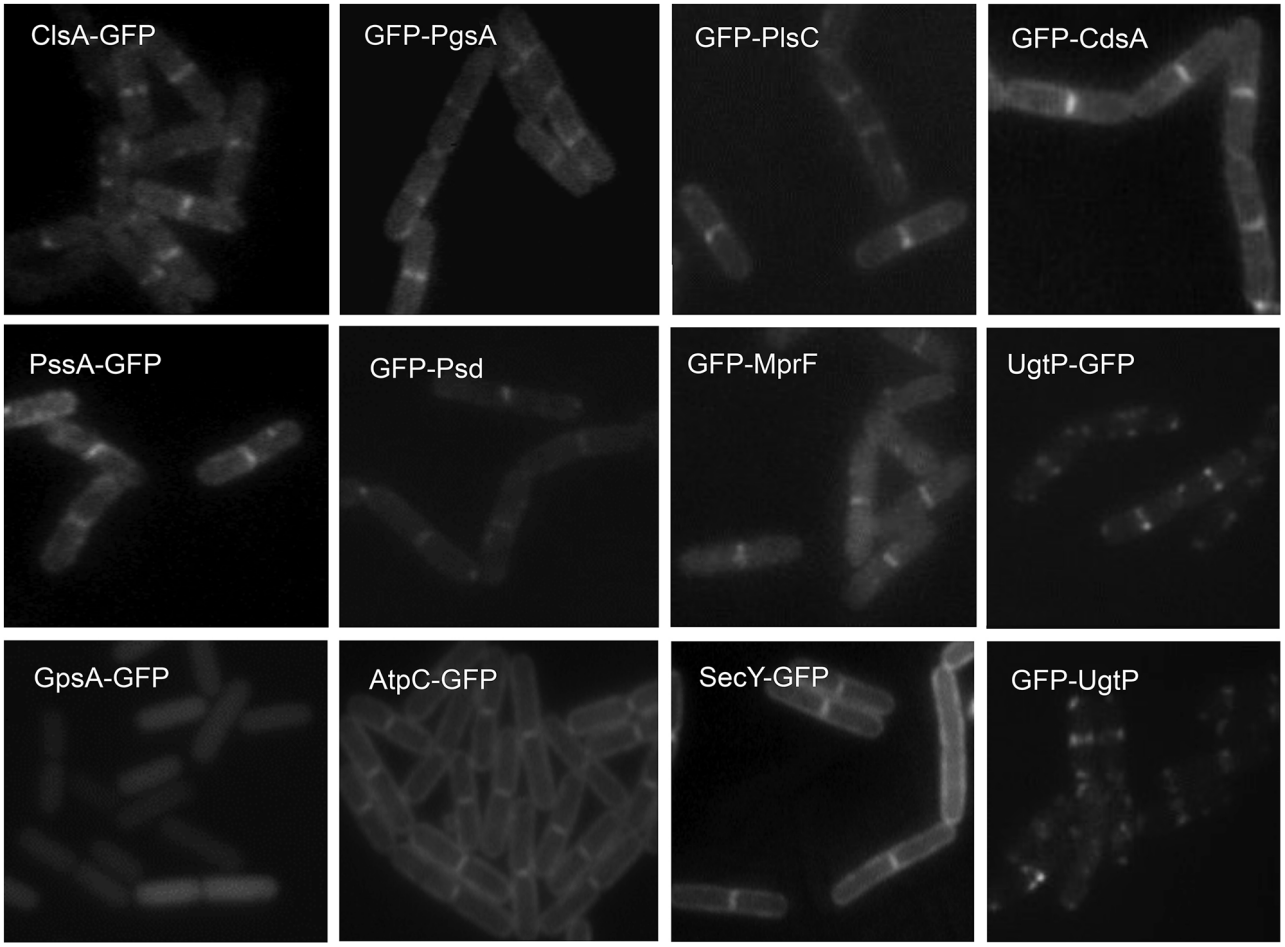
**Septal localization of lipid synthases in *B. subtilis* cells**. Typical florescence images from cells harboring *gfp* fusions in the *amyE* locus cultivated in sporulation medium (DSM) up to late logarithmic growth phase are shown. Green fluorescence from the GFP-fusions was detected by using a standard GFP(R)-BP filter unit. For the name of the enzymes and their catalytic activities refer to the figure of the biosynthetic pathway (**Figure 5B**). Panels are from Figures 4 and 5 of Nishibori et al. (2005), © American Society for Microbiology. GFP-fusions of ClsA and PssA are located in the septal membranes even when the corresponding genes are expressed from their natural promoters (see Figure 4 of Nishibori et al., 2005).

The septal and polar locations of specific phospholipids may be related to the subcellular location of the enzymes involved in their synthesis. In *E. coli*, a relationship between the location of CL (Mileykovskaya and Dowhan, 2009; Mileykovskaya and Margolin, 2012; see Cardiolipin and Other Anionic Phospholipid Domains) and that of CL synthase (ClsA) and phosphatidylglycerolphosphate synthase (PgsA; Tan et al., 2012; Dowhan, 2013) has yet to be reported. In *B. subtilis*, green fluorescent protein (GFP) fusions to ClsA and PssA, a PS synthase that catalyzes the committed step of biosynthesis of PE, are located in the septal membranes even when the corresponding genes are expressed at a low level from their natural promoters (Nishibori et al., 2005). Thus, ClsA is probably concentrated in the septal membranes under natural conditions thereby playing an important role in the septal localization of CL in *B. subtilis* (**Figure 3**). Localizing the enzymes involved in the synthesis of other lipids has yielded unexpected, interesting results. GFP fusions to several phospholipid synthases were localized to the septum in a thick, bright fluorescence band. These synthases include PgsA, PssA, Psd, which converts PS into PE, CdsA, which produces CDP-diacylglycerol, MprF, which transfers lysine to PG to produce lysylPG, and UgtP, which is responsible for glucolipid synthesis. The dot-pair distribution of UgtP corresponds to its role in regulating FtsZ assembly and hence differs from the distribution of the phospholipid synthases (see Sensing). The locations of these enzymes thus differ from the cytoplasmic location of GpsA, which catalyzes the production of G3P (Nishibori et al., 2005; **Figure 3**), and the uniformly distributed locations of the membrane proteins AtpC, a subunit of ATP synthase, and SecY (Matsumoto et al., 2012). It has also been shown that the enzymes for lipoteichoic acid (LTA) synthesis, LtaS and YfnI, are septally localized (Schirner et al., 2009; Matsuoka et al., 2011b), but DG kinase (DgkB), which converts DG that is produced in LTA synthesis into PA, is not localized (Matsuoka et al., 2011b).

How are the lipid synthases targeted to the septal membranes? These enzymes presumably have specific regions responsible either directly for their localization or indirectly via interaction with certain cell division proteins. Selective inactivation of putative targeting regions has shown that ClsA has regions of amphipathic α-helices at its COOH-terminus that are responsible for its septal localization (Kusaka et al., manuscript in preparation). PgsA has no such amphipathic α-helices at its COOH-terminus, suggesting that the system for its septal localization is different from that of ClsA. Many other proteins with amphipathic α-helices, including MinD (see Lipid–Protein Interactions), have membrane binding properties that depend on electrostatic interactions, *via* their net positive charges, and on hydrophobicity. In addition, the catalytic domains (or consensus sequences) of *B. subtilis* PssA (Saha et al., 1996b) and *E. coli* and *B. subtilis* PgsA (Usui et al., 1994; Matsumoto, 1997) have an amphipathic α-helix structure which allows them to access their hydrophilic substrates, serine and G3P, in the cytoplasm (Matsumoto et al., 2012). The septal location of the phospholipid synthases in *B. subtilis* cells implies that most phospholipids are synthesized at the septal membranes, and, given the septal location of PE and CL, it is likely that these newly synthesized lipids are somehow prevented from diffusing into the lateral membranes (Matsumoto et al., 2006).

### Chemical Basis for Generation of Lipid Domains

What then is the chemical basis for the generation of lipid domains? How do the lipid molecules form domains in membranes that are fluid? Both lipid–lipid interactions and lipid–membrane protein interactions are suspected to induce the formation of microdomains, which comprise at most some tens of molecules of a specific lipid (for a review, see Edidin, 1997). The following properties of phospholipid molecules may account for the formation of microdomains through lipid–lipid interactions.

Cardiolipin, which is a diphosphatidylglycerol, has a unique head group, with a tightly locked, surprisingly small configuration and only one negative charge (Haines and Dencher, 2002); this result contradicts the previous view reported in textbooks. The two phosphates in the head group trap a proton and are locked in a bicyclic array held together by the hydroxyl residue of the glycerol which connects the two halves. This small polar head group makes for a tighter packing of hydrophobic acyl chains between CL molecules by van der Waals force interactions than is found in lipids with larger polar head groups. It has also been suggested (Haines and Dencher, 2002) that interaction between adjacent head-groups creates a compact array of CL molecules, which becomes manifest in the presence of associated NAO arrays (Mileykovskaya et al., 2001).

Recently, a microtechnology for manipulating bacterial membrane curvature and quantitatively measuring its effect on the localization of CL in spheroplasts has been developed (Renner and Weibel, 2011). CL domains were localized to regions of high intrinsic negative curvature and MinD was found to be associated to the region of large negative curvature (see below). This localization of CL to membranes of highly negative curvature is ascribable to its cone-like molecular configuration (Gennis, 1989; Jouhet, 2013; see Cardiolipin and Other Anionic Phospholipid Domains). Polar localization of PA domains found in *E. coli*Δ*pgsA* cells lacking both CL and PG can also be ascribable to the cone-like configuration of PA molecule (Norris et al., 2002; Mileykovskaya et al., 2009).

Polar head groups of PG molecules interact by an extensive network of hydrogen bonds, ionic bonds, and coordination bonds between glycerol hydroxyls and the unesterified phosphate oxygen both in the anhydrous crystal and the hydrated gel state (Boggs, 1987; Pascher et al., 1987; Seddon, 1990). In fact, PG is probably segregated into distinct domains, in both *B. subtilis* and *E. coli* membranes, according to studies using pyrene-labeled phospholipids (Vanounou et al., 2003). This extensive and tight network of PG molecules may cause exclusion of CL molecules to produce patches of CL in the membranes (Mileykovskaya et al., 2001), since the small and tightly locked head group of CL cannot interact with PG.

The head group of the PE molecule has both a cationic amine residue and an anionic phosphate residue. Each amine and unesterified phosphate oxygen can participate in short distance intermolecular hydrogen bonds. The ethanolamine groups thus form a linkage between phosphorus groups of adjacent PE molecules and the tendency to be hydrated must be less than that of phosphatidylcholine, so producing a very compact, rigid head-group network of PE molecules (Elder et al., 1977; Hauser et al., 1981; Boggs, 1987; Seddon, 1990), and giving PE substantially higher *T*_m_ values than phosphatidylcholine which has an identical acyl chain structure. This compact head-group network of PE may well suffice to explain the formation of PE microdomains. Interactions with certain membrane proteins might then modify the lipid organization and further stabilize the head-group network, as suggested (Edidin, 1997). Polar and septal localization of PE in *B. subtilis* cells (Nishibori et al., 2005) is likely ascribable to its cone-like molecular shape (see Cardiolipin and Other Anionic Phospholipid Domains). For details refer to the previous review (Matsumoto et al., 2006).

The potential role for polyamines in lipid domain formation is largely unexplored although it has been suggested that polyamine binding to the acidic groups of phospholipids in membranes could lead to clusters forming (Schuber, 1989). Ionic interactions between polyamines and acidic phospholipids are strongest between the polyamine with the highest positive charges and the phospholipid with the highest content of negative groups (Yung and Green, 1986). Interaction with polyamines is believed to reduce the repulsive forces between negatively charged membrane components by bridging proteins and lipids and by shielding the surface charges (Schuber, 1989). The binding of ions such as calcium to anionic phospholipids can also result in domain formation (Haverstick and Glaser, 1987) and for references see Cannon et al. (2003), but, again, this is relatively unexplored.

## Transertion and Membrane Heterogeneity

### Heterogeneous Distribution of Envelope and Envelope-Associated Proteins

A heterogeneous distribution of proteins in the cell envelope of bacteria, despite their size, has been widely demonstrated (see reviews, Shapiro et al., 2002; Fishov and Norris, 2012b; Govindarajan et al., 2012). During the course of investigation of specific biological functions and processes, this heterogeneity has been revealed mainly by visualization with GFP-fusions and immunofluorescence to take the form of polar, patchy and helix-like distributions in the membranes. Examples of proteins localized to the septal regions include lipid synthases in *B. subtilis* (Nishibori et al., 2005; Matsumoto et al., 2012; Takada et al., 2014; described in Lipid Synthases and Membrane Domains). Reciprocally, ATP synthase and succinate dehydrogenase are at low levels in (or absent from) the mid-cell region at the onset of cell division in *B. subtilis*, which may reflect an association with lipid domains elsewhere that are rich in PG or other lipids rather than with lipid domains in the mid-cell region (i.e., the division site) where these lipids may be at low levels (Meredith et al., 2008). Recently, FloT and FloA, homologues of eukaryotic flotillin proteins found exclusively in lipid rafts along with proteins involved in signaling and transport have been localized to discrete microdomains in the membrane of *B. subtilis* (see Cardiolipin and Other Anionic Phospholipid Domains); significantly, these microdomains, which are likely to be present in many other bacteria, also contain other proteins involved in signal transduction and cell–cell communication such as the sensor kinase, KinC, and protein secretion such as SecY in *B. subtilis* (Donovan and Bramkamp, 2009; Lopez and Kolter, 2010; Bach and Bramkamp, 2013; Bramkamp and Lopez, 2015). Flotillins are believed to play a large part in maintaining the overall physical heterogeneity of the membrane since, in their absence, lipid-ordered domains coalesce (Bach and Bramkamp, 2013; Bramkamp and Lopez, 2015). Cytoplasmic membrane proteins located in the polar regions of *E. coli* cells include ProP, LacY, and MscS (Romantsov et al., 2010) and the MCPs (methyl-accepting chemotaxis proteins; Alley et al., 1992; Sourjik and Armitage, 2010) whilst proteins located at the sites of cell constriction in *E. coli* include the components of *trans-*envelope Tol-Pal complex, TolA, TolQ, and TolR (in the cytoplasmic membrane), the peptidoglycan-associated lipoprotein Pal, (anchored to the outer membrane), and TolB (a soluble periplasmic protein; Gerding et al., 2007).

Two systematic approaches have been taken to find proteins that are preferentially located in the poles. Using two-dimensional gel electrophoresis, Lai et al. (2004) identified proteins preferentially located in *E. coli* minicells and created a catalog of polar proteins; these included the inner membrane proteins MCPs, AtpA, AtpB, YiaF, and AcrA, the membrane-associated protein FtsX, and the outer membrane proteins YbhC, OmpW, Tsx, Pal, FadL, OmpT, and BtuB. In a complementary, cataloging approach, Li and Young (2012) used FLAG peptide-tagged Tar, a known polar MCP, to isolate by affinity capture those inner membrane vesicles that originated from the poles. These vesicles were enriched in over 30 proteins. Most were in or associated with the inner membrane and included Aer, PBP1B, TnaA, DcuA, PutP, TrxA, FliP, AccD, CpxA, FliC, RpsD, YcbC, GlnP, GroEL, MlaF, NarZ, YqjD, YniB, MCPs, CheA, YijP, PBP1A, SppA, LepB, NupC, and YiaF, although a few exceptions, TolC, Pal, and BamA, were in the outer membrane. In following up the latter catalog by fusing proteins to GFP and expressing the gene from their own promoters, Aer, TnaA, GroES (rather than GroEL), YqjD, and YiaF were found to form polar foci that were distinct from inclusion bodies, thereby suggesting that these proteins are located in polar membranes *in vivo*. Moreover, the MCPs and Pal figure in the polar membrane class in both cataloges, thus lending some confidence to the results. Extensions of approaches like these should identify the remaining proteins with polar preference using the full list of *E. coli* proteins, which includes as many as 1,133 predicted integral inner membrane proteins (Bernsel and Daley, 2009) and 503 peripheral, inner membrane-associated ones (Papanastasiou et al., 2013).

GFP-fusions and immunofluorescence have provided many examples of specific proteins in polar and helix-like distributions in the membranes of *E. coli* and *B. subtilis* cells; these proteins include MreB (as well as homologues MreBH and Mbl) and the Sec translocon (for the list and references see Table 1 in Fishov and Norris, 2012a). These results have, however, been questioned due to recent findings showing that artefactual distributions can be generated by high levels of expression. The translocon in *E. coli* includes the SecYEG translocon which can associate with SecDF–YajC–YidC and SecA to enable protein transport in a reaction that is stimulated by CL (Gold et al., 2010; Schulze et al., 2014). The GFP-fusions of SecA and SecY over-expressed using 1% xylose are helically distributed in *B. subtilis* (Campo et al., 2004) whilst expression from the native promoter Psec-*secA* gives a peripherally homogeneous and septal distribution of the fusion products in conditions in which careful high-level expression controls show helices (Carballido-Lopez et al., 2006-Supplementary Figure S6; Halbedel et al., 2014). This peripherally homogeneous distribution of SecA in *B. subtilis* is different from that of *E. coli* GFP-SecE which has a patchy subcellular distribution (Shiomi et al., 2006). Localization of FlAsH-tagged SecY and SecE in *E. coli* also shows quite different patterns from that of *B. subtilis* SecA, with helix-like (patchy), polar dots and a diffuse distribution in cells under conditions of no induction (Gold et al., 2010); that said, the cells still have higher levels of tagged products from the high copy number plasmid pBAD22, even in the absence of the inducer, compared with those from their native promoter on the chromosome, i.e., single copy. The translocon distributions in *B. subtilis* and *E. coli* also differ from that of the *Streptococcus pyogenes* translocon, which forms a single microdomain (Rosch and Caparon, 2005; Rosch et al., 2007) and that of *S. pneumonia* D39, expressed from the native chromosomal loci, which localizes dynamically to different places (Tsui et al., 2011). GFP-fusions of MreB have been shown to move, as fragmented discrete patches, perpendicular to the long axis of *B. subtilis* cells using highly sensitive time-lapse imaging (Dominguez-Escobar et al., 2011; Garner et al., 2011). This movement of MreB did not, as one would have expected, follow a helical pattern. Recent reports and reviews suggest that the distribution pattern reported for *E. coli* MreB mainly resembles that reported for *B. subtilis* MreB: a punctuated pattern with pairs of dots or small bands generally described as helical (van Teeffelen et al., 2011; Chastanet and Carballido-Lopez, 2012; Margolin, 2012; Errington, 2015). Indeed, MreB proteins form elongated filamentous structures when over-produced or when observed in late phases of growth. In virtually all older reports describing MreB localization, inducible, mostly over-expressed, GFP fusions were used, and observations were often made during the late exponential stage, when the structures were easier to visualize. This could be the case for other fusion proteins showing polar and helix-like distributions under highly expressed conditions. Thus, it is difficult at present to decide how many of the proteins do indeed form long-range, helical structures at the membrane, as noted by Margolin (2012).

### Lipid–Protein Interactions

**FIGURE 4:**
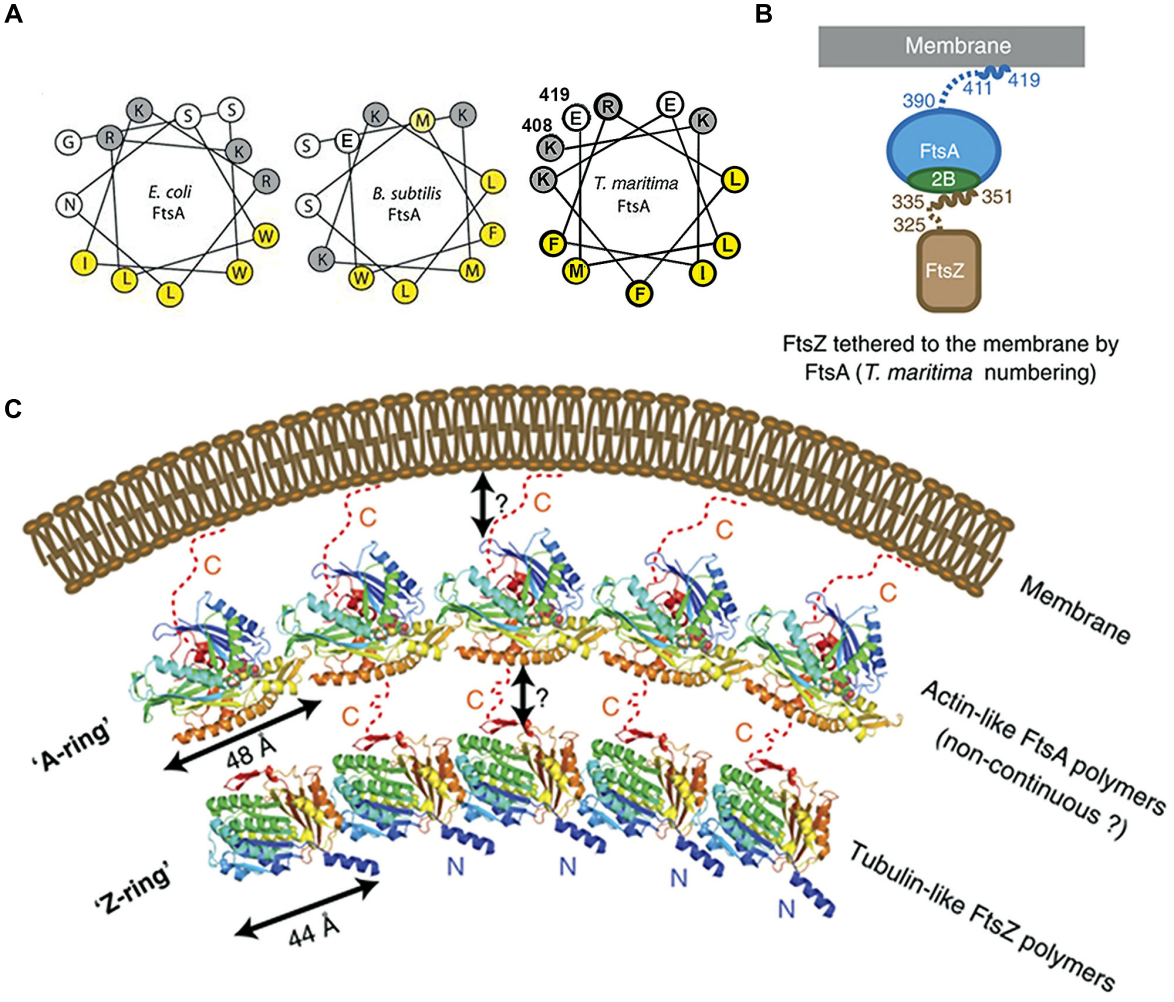
**Crucial role of the COOH-terminal amphipathic α-helix of FtsA in tethering FtsZ to the membrane. (A)** Helical wheel diagrams of the COOH-terminal domains of FtsA from *E. coli, B. subtilis* (Pichoff and Lutkenhaus (2005), © John Wiley and Sons with permission.) and *Thermotoga maritima* (prepared using Helixator-TCDB: www.tcdb.org/progs/helical_wheel.php). Hydrophobic residues are highlighted in yellow and negatively charged residues are highlighted in gray. **(B)** FtsA/FtsZ membrane-bound complex of *T. maritima.* **(C)** The Z-ring made of polymerized tubulin-like FtsZ may be attached to the membrane by an A-ring, made of actin-like FtsA polymers. The two cartoons are adapted from Szwedziak et al. (2012), © John Wiley and Sons with permission.

The last stage of transertion (see below) involves interaction of nascent proteins with the polar head groups of lipids followed by insertion into non-polar acyl-chain layer of the membrane. Proteins with a preference for a specific polar head group interact to form a specific domain in the membrane. Integral inner membrane proteins with specific lipid preferences could therefore be located in specific membrane domains (for the list of proteins see Table 2 in the review of Fishov and Norris, 2012a). In addition, peripheral membrane proteins that associate weakly with specific lipids could help form specific domains and, if these proteins bind other proteins, could bring them into the domain too. Among the peripheral membrane proteins, there are amphitropic proteins that have two apparent locations: one form of the protein is in the aqueous compartment of the cytosol whilst the other form is weakly associated with the cell membrane. Amphitropic proteins can be classified into three major categories (Johnson and Cornell, 1999), based on the way they associate with membranes. The first class (A) contains motifs with binding pockets for a lipid monomer, called lipid clamps. The second class (B) contains lipid covalent anchors embedded in the lipid bilayer; this class includes lipoproteins such as the major outer membrane lipoprotein (Braun’s lipoprotein), LolB, components of Bam complexes and RcsF with an *N-*acyl chain and a diacylglyceryl moiety at the NH_2_-terminus (Sankaran and Wu, 1994; Matsumoto, 2001; Suzuki et al., 2002; Okuda and Tokuda, 2011; Shiba et al., 2012; Konovalova et al., 2014). *E. coli* has 96 lipoproteins, 58% of which have completely unknown functions (Brokx et al., 2004). The third class (C) of amphitropic proteins contains amphipathic α-helices, which bind to a membrane by partitioning into the membrane bilayer such that the hydrophobic face of the protein is sequestered away from water, yet its polar face can contact the aqueous phase (Johnson and Cornell, 1999). The helix axis lies parallel to the membrane surface. The weak (reversible) interaction of the proteins with amphipathic α-helices plays an important role in regulation of their functions. Representatives of this class include a family of prokaryotic cytoskeletal proteins such as MreB, MinD, and FtsA, having an amphipathic α-helix at their NH_2_- or COOH-terminus (Szeto et al., 2003; Pichoff and Lutkenhaus, 2005; Salje et al., 2011; Szwedziak et al., 2012; **Figure 4**). Membrane anchoring with amphipathic helices has yet to be reported for any eukaryotic filament (Salje et al., 2011). It has been demonstrated recently that the nucleoid occlusion protein of *B. subtilis*, Noc, also associates with the cell membrane via an NH_2_-terminal amphipathic α-helix. It occludes assembly of the division machinery by simultaneous binding to DNA and the membrane (Adams et al., 2015). PgsA, *B. subtilis* PssA (Matsumoto, 1997), and the well-understood mammalian CTP: phosphocholine cytidylyltransferase (CCT) have an amphipathic α-helical structure in the middle of the protein (Cornell and Taneva, 2006). The amphipathic α-helices that were found in DnaA (Garner and Crook, 1996; Makise et al., 2001) are not involved in membrane association; instead, the results of membrane retention experiment obtained with various fragments of the protein indicated that the association is through a concerted interaction of distant residues forming a surface (Regev et al., 2012).

MreB is one of the key components of the bacterial cytoskeleton that lies just underneath the membrane and organizes the cell wall synthesis machinery. In *E. coli*, MreB has an amphipathic α-helix (an 11 amino acid residue sequence which includes two methionine, two phenylalanine, one leucine on one face of the helical wheel and three basic residues on the other face) at its NH_2_-terminus that is responsible for its location underneath the membrane. In gram-positive bacteria and the thermotrophic archaeum *Thermotoga maritima*, the counterpart of MreB has no such helix (Salje et al., 2011) and the membrane binding of *T. maritima* MreB is mediated by a small insertion loop that contains leucine and phenylalanine (residue 93 and 94), two other hydrophobic residues, the start methionine and a leucine (residues 1 and 2; Salje et al., 2011). MinD, plays a key role in division site selection by protecting the poles from aberrant positioning of FtsZ ring, and has a small amphipathic α-helix of 8–12 residues, termed the membrane targeting sequence or MTS, at its COOH-terminus that is conserved across eubacteria, archaea, and chloroplasts; this MTS is essential for association with anionic phospholipid-enriched membrane from which, in the case of *E. coli*, it can be easily detached by MinE at the appropriate time in the Min oscillation cycle (Raskin and de Boer, 1999; Szeto et al., 2002, 2003; Mileykovskaya et al., 2003; Vecchiarelli et al., 2014). The actin-like protein FtsA has an MTS that comprises a conserved COOH-terminal amphipathic α-helix of 13–14 residues (Pichoff and Lutkenhaus, 2005); FtsA polymerizes to form an “FtsA ring.” The tubulin-like protein FtsZ is tethered to FtsA through a COOH-terminal tail of 16 residues to polymerize to form an FtsZ ring underneath the A-ring (Szwedziak et al., 2012; **Figure 4**). Work on liposome division has shown the importance of the MTS in division. ATP is needed not only for the polymerization of FtsA and also for its attachment via its MTS to lipid monolayers and to vesicle membranes; this polymerization of FtsA caused vesicle shrinkage, consistent with the protein facilitating division both indirectly by interacting with FtsZ and directly by altering the membrane (Krupka et al., 2014). A fusion of FtsZ-YFP-mts was constructed with an MTS at its COOH-terminus to tether it directly to the membrane; this allowed Z-rings to assemble in multi-lamellar tubular liposomes and to generate a constriction force in the presence of GTP without the need for any other protein (Osawa et al., 2008). Uni-lamellar liposomes incorporating FtsA and FtsZ-YFP produced more natural Z-rings, which constricted liposomes and in some cases appeared to complete the division (Osawa and Erickson, 2013). The co-polymerization of FtsZ and FtsA is proposed to lead to bending, curvature and membrane constriction because the subunit repeat lengths of FtsZ and FtsA are different, being roughly 4 and 5 nm, respectively (Szwedziak et al., 2014; **Figure 4**).

### Membrane Curvature and Protein Location

In rod-shaped bacterial cells, negatively curved membranes characterize the poles and parts of the developing septum. In *B. subtilis*, it has been suggested that this curvature is sensed by DivIVA (Lenarcic et al., 2009; Ramamurthi and Losick, 2009), which is located at the septum and at mature poles and which is responsible for the polar location of the division inhibitor MinC/MinD via MinJ, a system different from the Min system of *E. coli* (Marston et al., 1998; Bramkamp et al., 2008; Patrick and Kearns, 2008). In cells with engineered curvature, DivIVA is indeed located preferentially in regions of high negative curvature (Renner et al., 2013; **Figure 2F**). The location of DivIVA depends on SecA although DivIVA does not contain a signal sequence (Halbedel et al., 2014). Instead, SecA is considered to act as a chaperone in the folding of DivIVA, which has essentially an α-helical structure with several coiled-coil helices that contribute to the curvature of the DivIVA tetramer (Halbedel et al., 2014). The crystal structure of this tetramer resembles the crescent shape of eukaryotic BAR domains which bind to curved membranes and also introduce curvature (Lemmon, 2008; Oliva et al., 2010; Mim and Unger, 2012). Enzyme I (EI), which is a part of the phosphoenolpyruvate-phosphotransferase system (PTS) responsible for the sensing and uptake of many extracellular sugars, is located in the poles of *E. coli* and *B. subtilis* due, it is proposed, to its affinity for negatively curved membrane, an affinity shared by DivIVA (Govindarajan et al., 2013). Conversely, SpoVM, a small peripheral membrane protein with an amphipathic α-helix, associates with the positively curved (convex) membrane surfaces of the forespore to form the spore coat complex with SpoVA, in the mother cell during sporulation (Ramamurthi, 2010; **Figure 2F**). Recently, it has been suggested that the actual amphipathic α-helix region of SpoVM (26 aa) is atypical; this region is short with only 13 residues (from 11 to 23**)**, contains only one positively charged residue (Arg) and three Gly residues, and inserts deeply into the membrane, unlike many amphipathic α-helix molecules that only insert shallowly into the membrane (Gill et al., 2015). The authors hypothesize that deep insertion of SpoVM into membrane, which involves extensive interactions with acyl chains to sense packing differences in differently curved membranes and which may involve cooperative interactions with other SpoVM proteins, drives its preferential localization onto slightly convex membranes surface such as the outer surface of the forespore.

### Transertion

In the transertion hypothesis, it is proposed that the coupled transcription-translation of genes encoding membrane proteins (1) structures the membranes, both physically and chemically, and (2) positions these genes close to the membrane (Norris and Madsen, 1995; Woldringh, 2002; **Figure 1**). Despite early evidence in its favor (Binenbaum et al., 1999), this hypothesis lacked further evidence to support it for long time. Recently, Libby et al. (2012) have shown that the synthesis of membrane proteins expression affects the position of chromosome loci in *E. coli* cells. They observed that two genetic loci, *lacY* and *tetA*, which encode membrane proteins, were rapidly shifted toward the membrane upon induction whilst the chromosomal locus for a cytoplasmic protein was not shifted; in addition, antibiotics that block transcription and translation prevented these shifts toward the membrane. More recently still, a radial contraction of the *E. coli* nucleoids was observed immediately after the addition of inhibitors of either transcription or translation, again consistent with transertion normally exerting an expanding force on the nucleoid (Bakshi et al., 2014). In the case of inhibition of transcription and hence transertion by Rifampicin, the eventual expansion of the nucleoid has recently been attributed to the penetration of the nucleoid by the ribosomal subunits (Bakshi et al., 2014; Sanamrad et al., 2014). That said, it should be noted that transcription is the essential source of supercoiling (particularly at high growth rates when 50–80% of active RNA polymerases are transcribing *rrn* genes) which is a major cause of nucleoid compaction; hence, inhibition of transcription initiation with Rifampicin results in RNA polymerase run-off, less supercoiling, and nucleoid expansion; this expansion can happen even after nucleoid compaction by chloramphenicol when transertion is absent (for a review, see Jin et al., 2013).

Over 1,100 genes distributed throughout the *E. coli* genome are predicted to encode integral inner membrane proteins (Bernsel and Daley, 2009) and therefore their expression could maintain the chromosome in an expanded and dynamic state, consistent with the transertion hypothesis. Significant numbers of ribosome and RNA polymerase copies have been shown by superresolution imaging to extend from the nucleoid to the cytoplasmic membrane (Bakshi et al., 2012), indicating that transertion exerts a direct radially expanding force on the nucleoid (Woldringh, 2002; Woldringh and Nanninga, 2006). In this study, however, few copies of RNA polymerase were found near the polar caps, indicating that transertion could not occur in the regions away from the nucleoid (polar caps) and therefore it might seem that the radially expanding force on the nucleoid of transertion could not be a direct axially expanding force. Such axial expansion might, however, result from the penetration of the nucleoid by the 30S and 50S subunits released from the 70S ribosomes following treatment with inhibitors of translation (Bakshi et al., 2014; Sanamrad et al., 2014). It should be noted that although less than 15% of *E. coli*’s ribosomes are reported to be available to participate in transertion (Bakshi et al., 2012; Gahlmann and Moerner, 2014), this figure does not necessarily reflect the percentage of the ribosomes that are actually engaged in translation. A higher percentage is likely to be available in *C. crescentus* where genes and mRNA have been found to be close to one another (Llopis et al., 2010).

There are plenty of polar membrane proteins as noted in the previous section (see Heterogeneous Distribution of Envelope and Envelope-Associated Proteins). Transertion is unlikely to be an important factor in directly controlling their location since, firstly, RNA polymerase is relatively scarce near the polar caps and, secondly, the Sec machinery responsible for inserting integral membrane proteins into membranes is not specifically located in the poles in either *B. subtilis* (Carballido-Lopez et al., 2006-Supplementary Figure S6) or *E. coli* (Shiomi et al., 2006; Gold et al., 2010; see Heterogeneous Distribution of Envelope and Envelope-Associated Proteins). Thus, some integral membrane proteins (e.g., the MCPs) are inserted into the cytoplasmic membrane around the nucleoid via the Sec machinery and then freely migrate in the membrane to the polar regions (Shiomi et al., 2006). One possibility is that integral membrane proteins such as the MCPs are excluded from the transertion domains around the chromosome because they have no affinity for the lipids in which those domains are enriched. The reason for this is that transertion may create a problem if it leads to the formation of an array of MCPs of the wrong size in the wrong place. Another, recently proposed, possibility is that polar localization of the diffused MCPs occurs independently of the phospholipid composition of the cytoplasmic membrane and is not be dictated by the curvature of the cell poles, instead, MCPs interact with components of the trans-envelope Tol-Pal complex which restricts the diffusion of MCP arrays (Santos et al., 2014; see Transertion Problems).

In the original transertion hypothesis, the structuring of the membrane and the tethering of genes was restricted to those genes encoding proteins inserted into or through membranes. Although the role of the former class of proteins (i.e., integral membrane proteins) is easy to imagine, the latter class (i.e., secreted and exported proteins) is also of interest. For example, there could be a role in transertion for Braun’s lipoprotein if the lipid modification at the NH_2_-terminus of the lipoprotein were to occur before the synthesis of the rest of the protein. Moreover, the transertion hypothesis could be extended to genes encoding peripheral membrane proteins such as SeqA (Slater et al., 1995; Shakibai et al., 1998) and DnaA in *E. coli* (Regev et al., 2012) and Noc in *B. subtilis* (Adams et al., 2015).

Finally, it has been proposed recently on entropic grounds that transertion enables ribosomal subunits to penetrate the nucleoids and to initiate the more general process of co-transcriptional translation (Bakshi et al., 2014). Co-transcriptional translation would then protect nascent mRNA (Deana and Belasco, 2005; Deneke et al., 2013) and prevent the backtracking of RNA polymerases (McGary and Nudler, 2013), thereby giving transertion a key role in the optimization of transcription and translation (Bakshi et al., 2014). In the context in which two chemically identical chromosomes are in competition for the transcriptional and translational apparatuses, such optimization constitutes a positive feedback that could underpin differentiation (Norris and Madsen, 1995; Norris and Amar, 2012).

## Hyperstructures

A hyperstructure is an assembly of elements (such as genes, RNA, proteins, small molecules and ions) that performs a function and that constitutes a substantial proportion of the cellular material (Norris et al., 2007a; Saier, 2013; Meyer et al., 2014). The accumulating evidence for their existence allows them to be classed in several ways (Norris et al., 2007b). In the case of the membrane, the best known include those involved in motility (such as the MCPs and flagella), lactose metabolism, energy generation, and cell division. It has been further proposed that a dialog between hyperstructures determines the phenotype of the cell (Norris et al., 2014a). Here we focus on some of the hyperstructures involved with the membrane.

### Transertion Hyperstructures

The coupled transcription-translation-insertion of membrane proteins (transertion) has been proposed to tether networks of nascent proteins, mRNAs and genes to the cytoplasmic membrane thereby attaching DNA dynamically to the bacterial cell envelope (Norris, 1995; Norris and Madsen, 1995; Woldringh, 2002; **Figure 1**). This means that the high level expression of a gene encoding a membrane protein could lead to the formation of a domain in the membrane enriched by the nascent proteins, possibly specific lipids, and translocon complexes, along with an adjacent, highly structured cytoplasm to give a transertion hyperstructure (Norris, 1995; Norris and Madsen, 1995; Binenbaum et al., 1999; Woldringh, 2002).

The *lac* operon is an obvious candidate for giving rise to a transertion hyperstructure. In *E. coli*, full induction of this operon has been calculated to give 23 RNA polymerases on *lacZ* and 5 or 6 on *lacY* and *lacA* with the ensemble of the nascent, full length and decaying mRNAs being translated by several hundred ribosomes (Kennell and Riezman, 1977). *lacY* encodes the membrane-bound permease with an average of four ribosomes calculated as translating the corresponding full length mRNA (Kennell and Riezman, 1977). LacZ-encoding mRNA has been shown to remain close to the *lacZYA* locus, consistent with both transcription and translation occurring in the same place (Llopis et al., 2010). Expression of the *lac* operon brings the locus closer to the membrane and this movement depends on the presence in the operon of *lacY*, consistent with transertion (Libby et al., 2012). Hence, calculations and experiments suggest that a transertion hyperstructure exists that comprises the *lac* genes dynamically attached to tens of nascent mRNAs and to 100s of ribosomes and the nascent enzymes some of which would be inserted into the membrane. This does not, of course, mean that the Lac proteins once synthesized remain within the transertion hyperstructure. Indeed, a tagged version of LacY – albeit expressed from a plasmid – was localized to the poles (Romantsov et al., 2010).

RNA degradation may also play a part in the dynamics of transertion hyperstructures. Using epifluorescence microscopy and molecular dynamics simulation, RNase E, the backbone of the RNA degradosome, was shown to diffuse over the entire inner membrane of *E. coli* to form, along with other proteins, short-lived hyperstructures (Strahl et al., 2015). The existence of these hyperstructures depends on the presence of the RNA substrate (Strahl et al., 2015) so they may be considered as *functioning-dependent* structures, that is, structures that form due to their activity (Norris et al., 2007a). It should be noted that such separation of the degradosomes from the sites of transcription would favor nascent transcripts being translated by polyribosomes, that is, would favor the coupling of transcription and translation over the separation of these processes (Strahl et al., 2015).

### Transertion Problems

Transertion is not only a likely solution to many of the problems that confront cells but also a source of potential problems. One problem would occur if a transertion hyperstructure were to determine, inappropriately, the size, position or composition of another class of hyperstructure. Consider, for example, the importance in signal transduction of the distribution of the MCPs and related proteins into many small clusters or into one giant cluster (Bray et al., 1998). These distribution confer different sensitivities but this relationship could be lost if transertion were to dominate by creating a large chemosignaling hyperstructure. In *E. coli*, part of the solution may be that the genes that encode chemotaxis proteins are located on the chromosome so that their transertion associates them with another major hyperstructure, that of the flagellum; it is then conceivable that differences in the affinities of the chemotaxis and flagellar proteins for the chemical or physical properties of lipids are responsible for the chemotaxis proteins relocating from the flagellar hyperstructure(s) to the poles (Cabin-Flaman et al., 2005). Consistent with this, Tar-GFP does diffuse away from the sites where this MCP is synthesized to the poles (Shiomi et al., 2006). It has been recently found that this polar localization of the diffused MCPs does not depend on either the phospholipid composition of the cytoplasmic membrane or the curvature of the cell poles; instead, MCPs interact with components of the trans-envelope Tol-Pal complex which restricts the diffusion of MCP arrays (Santos et al., 2014). The Tol-Pal complex is also part of the cell division hyperstructure, to which it is recruited by FtsN so as to play a role in the invagination of the outer membrane during division (Gerding et al., 2007).

A second problem would arise if the lipid preferences of the constituents of the transertion hyperstructure were to menace the planar, bilayer structure of the cytoplasmic membrane. The reality of this danger is evident from the proteo-lipid structures that result from the overproduction of peripheral and integral membrane proteins (Weiner et al., 1984; Arechaga et al., 2000; van den Brink-van der Laan et al., 2003; Eriksson et al., 2009). In mitochondria, the bioenergetic supercomplexes are indeed in specific membrane structures whilst in bacteria such as *E. coli*, fluorescent fusions suggest that the complexes are spatially dispersed in mobile 100–200 nm domains containing 10s–100s of complexes (Erhardt et al., 2014; Llorente-Garcia et al., 2014). A possible solution would be for an abundant structure such as the ATP synthase to have subunits with complementary lipid preferences (Arechaga et al., 2000) since even overproduction of all eight subunits results in morphological changes (von Meyenburg et al., 1984). Extending this argument, one might expect different proteins to have preferences for different lipids, which may help explain why cells have so many different lipids. An alternative or complementary solution would be the creation by a large, dynamic hyperstructure of many mobile domains with an affinity for ATP synthase.

Finally, problems due to the formation of an inappropriate transertion hyperstructure might be avoided by reducing the time during its synthesis in which the nascent protein interacts with the membrane; this might be achieved if, for example, evolution were to have selected for the membrane-interacting sequences (such as amphipathic helices) to be located at the COOH-terminus rather than at the NH_2_-terminus.

### Sensing

The membrane is the final frontier between the cell and its environment so membrane-based hyperstructures are in the right place to sense and to respond to environmental changes. This task is not trivial. Not only do new mRNAs and proteins have to be made but many existing mRNAs and proteins must be degraded. There are, for example, probably over a 1000 membrane proteins in *E. coli* (Bernsel and Daley, 2009) and a similar number in *B. subtilis* (Hahne et al., 2008; Otto et al., 2010; van Dijl et al., 2012) and the functioning of these proteins must be coordinated; this task is particularly difficult when major changes in the protein composition of the membrane must occur as during changes in growth conditions. On the entry of *B. subtilis* to stationary phase, the levels of the many proteins that increase include those involved in the uptake of glycerol, ribose, lactate, nucleoside, succinate, fumarate and zinc whilst the levels of those that decrease include transporters of malate, Fe^3+^ citrate, Fe^3+^ hydroxamate, and hydroxymethylthiamine (HMP)/thiamine transport (Otto et al., 2010). In *E. coli* cells supplemented with glucose, which is the preferred carbon and energy source, the transcriptional levels of the many genes that increase include those involved in the import of polyamines, inorganic phosphate and Mg^2+^ whilst the levels of those that are repressed (in other words, upregulated upon glucose–starvation), are of transporters and periplasmic receptor proteins related to the import of alternative carbon and carbon–nitrogen sources, which include amino acids, carbohydrates, lactate, glycerol, peptides, dipeptides, and nucleotides (Gutierrez-Ríos et al., 2007). This transcriptome pattern is consistent with the consequences of carbon catabolite repression exerted by glucose (Saier et al., 1996). The phosphoenolpyruvate-dependent PTS controls the uptake of a large number of energetically preferred sugars. It comprises proteins, EI and HPr, which are common to all substrates, as well as sugar-specific permeases, enzymes II (EIIs). Interestingly, EI and HPr are mainly located near the poles of *E. coli* cell (Patel et al., 2004; Lopian et al., 2010; Govindarajan et al., 2012). Upon addition of the sugar to the growth medium, HPr is phosphorylated by EI; HPr-P produced is released from the polar membranes and distributes in the cell, though EI remains located near the poles of negatively curved sites (Govindarajan et al., 2013; see Membrane Curvature and Protein Location). A fraction of HPr-P gets near the membrane to phosphorylate the permeases, allowing them to transport the sugars into the cell and to phosphorylate them (Govindarajan et al., 2012).

Common affinities for molecules and ions within and between hyperstructures may help in this task of coordination. These molecules include polyamines, poly-(R)-3-hydroxybutyrate, polyphosphate and, of course, lipids (Norris et al., 2014b). Polyamines, for example, bind to nucleic acids, ribosomes and porins, and stimulate the synthesis of around 300 proteins in *E. coli* that include the sigma factors RpoS, FecI, RpoN, and related RNA polymerase omega subunit RpoZ, Cya, Cra, Fis, H-NS and SpoT (Igarashi and Kashiwagi, 2006). These multiple actions may allow polyamines to play an important, coordinating, role in the survival of *E. coli* in the transition to stationary phase (Terui et al., 2012; Norris et al., 2014b) and during osmotic changes (Munro et al., 1972).

**FIGURE 5:**
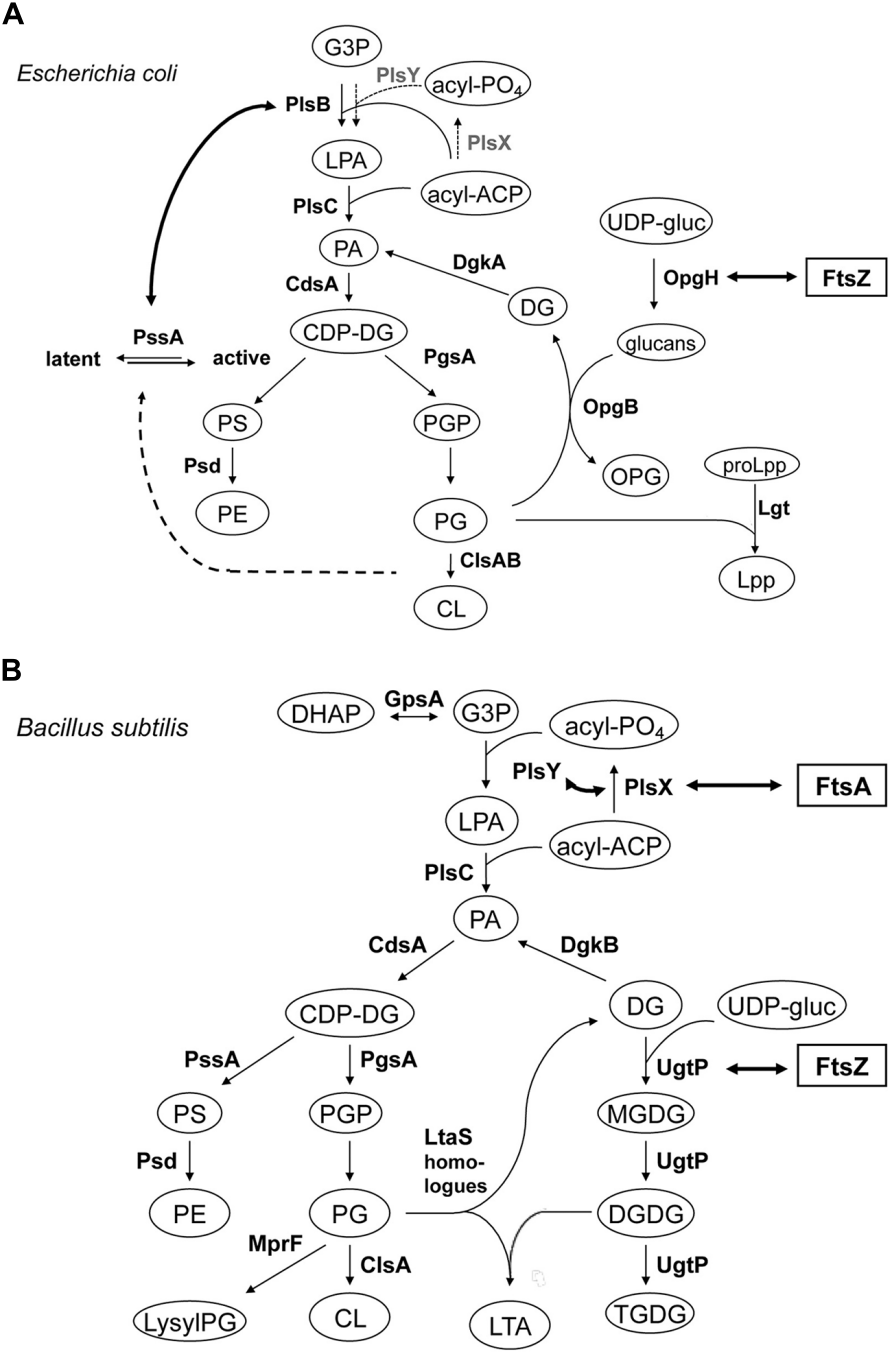
**Lipid biosynthetic pathways and interactions of phospholipid synthases and proteins involved in cell division. (A)** Phospholipid biosynthetic pathway in *E. coli.* The gene product catalyzing each step is indicated. Abbreviations used are: G3P, glycerol-3-phosphate; LPA, lysophosphatidic acid; PA, phosphatidic acid; acyl-PO_4_, acylphosphate; acyl-ACP, acyl acyl-carrier protein; CDP-DG, (d)CDP-diacylglycerol; PS, phosphatidylserine; PE, phosphatidylethanolamine; PGP, phosphatidylglycerophosphate; PG, phosphatidylglycerol; CL, cardiolipin; OPG, osmoregulated periplasmic glucans (or MDO); UDP-gluc, UDP-glucose; DG, diacylglycerol; Lpp, major outer membrane lipoprotein; proLpp, prolipoprotein. Trace activities of PlsX-PlsY for LPA synthesis in *E. coli* (Lu et al., 2006/Hara et al., 2008) were indicated with thin letters and dotted arrows, different from those of the major pathway in *B. subtilis*. For Cls homologues, ClsA and ClsB, refer to Tan et al. (2012). Interaction of OpgH (a glucosyltransferase) and FtsZ indicated with thick arrow is from Hill et al. (2013); see Sensing). Activation of phosphatidylserine synthase, PssA, with acidic phospholipids indicated with thick dotted arrow is adapted from Shibuya (1992) and Matsumoto (1997). Interaction of PlsB and PssA is from Gully and Bouveret (2006), but to avoid apparent complexity the interactions between PlsB, ACP and YbgC, an Acyl-CoA thioesterase, shown by the authors, are not depicted. **(B)** Biosynthetic pathways for phospholipids, glucolipids and LTA in *B. subtilis*. Abbreviations used are: lysylPG, lysylphosphatidylglycerol; MGDG, monoglucosyldiacylglycerol; DGDG, diglucosyldiacylglycerol; TGDG, triglucosyldiacylglycerol; LTA, lipoteichoic acid; DHAP, dihydroxyacetone phosphate. For other abbreviations refer to (A) for *E. coli*. The pathway is adapted and compiled from Jerga et al. (2007) and Matsuoka et al. (2011b). For LtaS homologues (YflE, YfnI, YqgS and YvgJ) refer to Gründling and Schneewind (2007), Matsuoka et al. (2011b) and Hashimoto et al. (2013). Interactions of FtsA and PlsX, and PlsY, both indicated with thick arrows, are from Hara et al. (2008) and Takada et al. (2014), respectively, but to avoid apparent complexity the interaction of PlsX with EzrA, DivIA and others, shown by the authors, are not depicted. Interaction of UgtP (a glucosyltransferase) and FtsZ indicated with thick arrow is from Weart et al. (2007) and Chien et al. (2012); see Sensing).

Similar global regulatory roles can be ascribed to CL and an increase in CL levels has been proposed as a general physiological response that protects microorganisms from lysis due to osmotic stress (Catucci et al., 2004) and, in line with this, an increase in osmolality leads to transcriptional activation of the *cls* gene in *E. coli* (Romantsov et al., 2007). This increase in CL and its distribution to the poles was correlated with the polar distribution of the osmosensory transporter ProP which actively transports osmo-protectants into the cell (Mileykovskaya, 2007; Romantsov et al., 2007). In such coordination of lipid metabolism with the environment, the formation of a hyperstructure containing membrane components may help. Acyl carrier protein (ACP), a small protein of 9 kDa, interacts with diverse proteins associated with many biosynthetic pathways, including enzymes involved in synthesis of fatty acid in the cytoplasm as well as enzymes on or in the membrane involved in phospholipid or LPS synthesis; the latter assocations could account for the partial localization of ACP with *E. coli* membranes. Two enzymes involved in phospholipid synthesis, PlsB, a *sn*-glycerol-3-phosphate acyltransferase and PssA, a PS synthase, have been shown to interact with ACP and YbgC, an acyl-CoA thioesterase involved in fatty acid synthesis, using a tandem affinity purification method (Gully and Bouveret, 2006). *E. coli* PssA, which has a preference for acidic phospholipids, exists as both a membrane-associated active form and a cytoplasmic latent form and is at the heart of a cross-feedback regulation of the synthesis of zwitterionic (PE) and acidic phospholipids (Shibuya, 1992; Okada et al., 1994; Saha et al., 1996a; Matsumoto, 1997; Rilfors et al., 1999; **Figure 5A**). Bacterial two-hybrid analysis has shown that the enzymes in the phospholipid synthetic pathway, PlsB-PssA and ACP, form a complex in/on the inner membrane and further that YbgC, a fatty acid synthesis enzyme, and PlsB, form a complex in association with ACP (Gully and Bouveret, 2006). Thus, physical interactions between many of the enzymes responsible for fatty acid synthesis and phospholipid synthesis could form a hyperstructure to help coordinate lipid metabolism.

Sensing changes in nutrient availability entails bacteria transmitting this information to flagella hyperstructures to perform chemotaxis (see Transertion Problems) and to the division apparatus to regulate their size, grow faster and become larger when they are grown in nutrient-rich media. UgtP, a glucosyltransferase responsible for the synthesis of glucosylated diacylglycerols, one of which is the anchor of LTA synthesis, is localized, in a dot-pair structure similar to that of the open ring of FtsZ, to the division site in *B. subtilis* (Nishibori et al., 2005). UgtP is a division inhibitor, which prevents assembly of FtsZ and which results in an increase in the length of the cells under nutrient-rich conditions (Shiomi and Margolin, 2007; Weart et al., 2007; **Figure 5**). It has been proposed that UDP-glucose acts as a proxy for nutrient availability and modulates the equilibrium between the UgtP–UgtP oligomer and the UgtP-FtsZ complex thereby serving as a molecular rheostat to help ensure that cell size is precisely co-ordinated with growth rate and nutrient availability (Chien et al., 2012). OpgH, a glucosyltransferase, is an integral membrane protein in the inner membrane of *E. coli* that is involved in the synthesis of osmoregulated periplasmic glucans, OPG (MDO); recently, OpgH has been localized to the division ring and shown to inhibit FtsZ assembly in the presence of UDP-glucose so as to delay the timing of cell division (Hill et al., 2013; **Figure 5A**). These two, very different, bacteria employ unrelated glucosyltransferases – a peripheral membrane enzyme for LTA synthesis in *B. subtilis* and an integral inner membrane enzyme for OPG synthesis in *E. coli* – that both have the second, “moonlighting,” function of coupling nutrient availability to cell division by regulating FtsZ assembly (Hill et al., 2013). Disruptions of the moonlighting function of UgtP not only affect cell length: *ugtP* null mutants, which lack glucolipid products of the UgtP-catalyzed reaction, membrane glucolipids, are rounder (Price et al., 1997) and abnormally bent and distended (Lazarevic et al., 2005; Matsuoka et al., 2011a). Moreover, the extracytoplasmic function (ECF) σ factors, σ^M^, σ^V^, and σ^X^ are constitutively activated (Salzberg and Helmann, 2008; Matsuoka et al., 2011a; Hashimoto et al., 2013), consistent with glucolipids directly influencing anti-σ^M^ and anti-σ^V^ factors by stabilizing conformations that sequester the respective ECF σ factors (Seki et al., 2015).

A two-hybrid-based investigation of interactions between the components of cell division machinery (27 proteins) and the enzymes involved in lipid synthesis (12 enzymes) in *B. subtilis* has revealed that FtsA interacts with PlsX (Takada et al., 2014). PlsX is an acyl-ACP:phosphate acyltransferase that synthesizes acyl-phosphate, an essential substrate for lysophosphatidic acid (LPA) production by PlsY, an acyltransferase that uses acyl-phosphate for the first acylation of *sn*-glycerol-3-phosphate (G3P); these are novel enzymes in a unique LPA synthesis pathway found in prokaryotic but not in eukaryotic cells (Lu et al., 2006; Paoletti et al., 2007; Yoshimura et al., 2007, and Hara et al., 2008; **Figure 5B**). *In vivo* cross-linking shows that PlsX interacts with cell division proteins (EzrA, DivIVA), cytoskeletal proteins and several metabolic enzymes, in addition to FtsA. PlsX is in a punctuated pattern in the peripheral membrane and is present at potential division sites independently of FtsA and FtsZ. Inactivation of PlsX leads to aberrant Z-ring formation. Thus, the key enzyme for phospholipid metabolism interacts with the cell division machinery in order to complete septum assembly (Takada et al., 2014). Although PlsX interacts with PlsY, many other enzymes involved in phospholipid synthesis, which localize in the septal region (Nishibori et al., 2005), do not interact with it, except MprF that synthesizes lysylPG (Takada et al., 2014). Depletion of PlsX leads to the cessation of both fatty acid and phospholipid biosynthesis (Paoletti et al., 2007; Yao and Rock, 2013).

### Cell Cycle

Transertion may help provide solutions to the problems of coupling growth to the cell cycle, of triggering chromosome replication, and of coupling chromosome segregation spatially and temporally to cell division. The first problem, the way in which growth is coupled to the cell cycle, has been a mystery that has lasted for over half a century (Schaechter et al., 1958; Osella et al., 2014). Many different metabolic processes occur during growth and these processes are needed for the synthesis of RNA, proteins and lipids. These processes come together in transertion, which is therefore well-placed to integrate metabolic information (Fishov and Norris, 2012b). Moreover, transertion is a primary determinant of the structure and composition of the membrane, which is central to the initiation of DNA replication, segregation and cell division, as well as to the structure of the chromosome, which is kept in an expanded state (Woldringh et al., 1995; Libby et al., 2012). Hence transertion is also well-placed to couple growth to the cell cycle. Consistent with this, major effects of transertion on the microviscosity of the membrane have been shown via inhibition of transcription and translation (Binenbaum et al., 1999). The second problem, the nature of the mechanism that initiates chromosome replication, is another long-standing mystery (Eliasson and Nordström, 1997; Wang et al., 2011). This mechanism in *E. coli* is generally attributed to the relative proportions of the DnaA ‘initiator’ protein in the ATP-DnaA form, which is active in initiation, and the ADP-DnaA, which is inactive (Castuma et al., 1993). The reasons to invoke lipids in a DnaA-based control of initiation include the involvement of unsaturated fatty acids in initiation (Fralick and Lark, 1973), the promotion of formation of ATP-DnaA *in vitro* by anionic phospholipids (Castuma et al., 1993) and the association of 10% of the DnaA in a cell with the membrane (Regev et al., 2012). Given its role in determining membrane dynamics, transertion may well lie at the heart of the initiator mechanism and act via DnaA and, more generally, via promoting strand separation (Norris and Amar, 2012).

The third problem, the coupling between chromosome segregation and cell division, is essentially a membrane problem in the sense that the cell must arrange for the membrane to invaginate and separate segregated chromosomes. One role for transertion may be to create membrane domains in a particular physico-chemical state around the segregating chromosomes such that a different, ‘septal’ domain forms by default between them (Norris, 1995; Woldringh, 2002; Norris et al., 2004). This septal domain might then generate tubular or other structures that would recruit and activate division proteins. This hypothesis is supported by evidence for cell cycle variations in the lipid composition of *E. coli* (Mozharov et al., 1985) and in the spatial distribution of the lipids of *Micrococcus luteus* (Welby et al., 1996), for the CL-rich domains at the division sites of *E. coli* (Mileykovskaya and Dowhan, 2000; Koppelman et al., 2001) and *B. subtilis* (Kawai et al., 2004), and the septal location of the polyunsaturated fatty acid eicosapentaenoic acid of *Shewanella livingstonensis Ac10* (Sato et al., 2012). Significantly, distinct domains appear around and between the segregating chromosomes at a very early stage of the cell cycle in *E. coli* and these domains disappear when translation – and hence transertion – is abolished (Fishov and Woldringh, 1999). Division is inhibited by the Min system and lipids are again important (Suefuji et al., 2002; Kretschmer and Schwille, 2014). The NH_2_-terminal domain of MinC perturbs the interactions between FtsZ monomers within an FtsZ polymer, while the COOH-terminal domain perturbs the lateral association of FtsZ protofilaments as well as interacting with MinD, for references see Kretschmer and Schwille (2014); MinD is a peripheral membrane-binding protein with an ATPase activity that is activated by MinE and that triggers the detachment of MinD from the membrane to prevent MinCD from inhibiting division; MinE may self-assemble on the membrane via an N-terminal helix that acts as an MTS. The idea is that, in *E. coli*, MinD binds cooperatively to the membrane at one pole with MinE forming an “E-ring” on the rim of this MinD zone that induces its disassembly leading to the diffusion of MinD and MinE and repetition of this assembly disassembly process at the other pole; MinC follows these oscillations of MinD and MinE to inhibit division (Rowlett and Margolin, 2013; Zheng et al., 2014 and references therein). *In vitro* and *in vivo*, MinD interacts with anionic lipids, which determine its distribution (Mileykovskaya et al., 2003), as does MinE (Vecchiarelli et al., 2014). In the case of the *E. coli* MinD introduced into *B. subtilis*, MinD makes spirals that coincide with the spirals of what are probably anionic phospholipids (Pavlendova et al., 2010). Moreover, MinD can convert phospholipid vesicles into tubes (Hu et al., 2002), consistent with the possible importance of phospholipid structures in nucleating division (Norris et al., 2004; Li et al., 2011). Finally, in spherical *E. coli* cells, FtsZ is associated with intracellular membraneous structures and MinD accumulates and oscillates between the places where these structures form (Bendezú and de Boer, 2008).

The 2 min or *dcw* cluster includes 16 genes, such as *ftsZ*, involved in peptidoglycan synthesis and cell division, and the promoter at the start of the cluster probably contributes to the transcription of the whole cluster, leading to the synthesis of a quite long mRNA (Hara et al., 1997; Mengin-Lecreulx et al., 1998; Vicente et al., 1998). Transertion of this cluster may therefore be an important factor in the spatio-temporal control of division. Transertion structures not only the membrane but also the cytoplasm thereby raising the possibility that chromosome segregation is accompanied by a phase separation in the cytoplasm that affects membrane dynamics and drives cell division. Hence, it is significant that phase separation of dextran and polyethylene glycol within giant vesicles leads to tubes of membrane forming at their interface (Li et al., 2011).

If transertion from the *dcw* cluster is indeed important, what is the consequence of having transertion from two clusters (one per segregating chromosome)? Are the lipid preferences of the secretion machinery different from those of the proteins encoded by this cluster so that the domain-producing potential of transertion is reduced? In bacterial L-forms, which lack a cell wall, it might be supposed that the requirement for many of the *dcw* genes would be reduced and less transertion would occur. This would be consistent with the findings that, in L-forms of *E. coli*, some of the genes were mutated (Siddiqui et al., 2006) and the levels of FtsZ were fivefold lower (Onoda et al., 2000) whilst in an L-form of *B. subtilis* FtsZ could be eliminated altogether (Leaver et al., 2009).

## Discussion

The different ways of making membrane domains include lipid–lipid, lipid–protein, protein–protein, and polyamine–lipid interactions. By integrating these interactions, transertion plays a central role in the organization of the bacterial cell. It might even be argued that transertion helps provide a conceptual context for thinking about a plethora of intracellular events, structures and processes including the regulation and execution of the cell cycle, hyperstructure dynamics and the origins of life. In 1989, the term ‘nucleoid occlusion’ was coined to describe the inhibitory effect of the nucleoids on cell division (Cook et al., 1989) and it was suggested that nucleoid occlusion “may be related to a transcription or translation activity of the nucleoid” (Mulder and Woldringh, 1989). A few years later, it was proposed that the coupled transcription-translation-insertion of nascent proteins into and through membrane (i.e., transertion) is the mechanism responsible for nucleoid occlusion (Norris, 1995; Norris and Madsen, 1995; Woldringh, 2002). In this proposal, transertion creates membrane domains at the right times and in the right places not only to control chromosome replication, segregation and cell division but also to create the positive feedback needed for differentiation (Norris, 1995; Norris and Madsen, 1995; Binenbaum et al., 1999; Norris et al., 2004; Norris and Amar, 2012). Recently, it has been suggested that the role of transertion in nucleoid occlusion is complemented by the recruitment of Noc nucleoprotein complexes and associated DNA to the membrane (Adams et al., 2015).

Transertion can generate hyperstructures (Norris et al., 2007a; Llopis et al., 2010). Such transertion hyperstructures form a large class that, along with the other classes of hyperstructures, constitute the bacterial cell. The exact conformation of transertion hyperstructures is unknown but could be very important in bacterial cell physiology if, as proposed, interactions between hyperstructures were to be the primary determinants of the phenotype (Norris et al., 2007a). For example, interactions based on the condensation and decondensation of ions (Manning, 2007) might enable transertion hyperstructures containing membrane domains and/or linear assemblies of macromolecules to drive the cell cycle (Norris and Amar, 2012) whilst the kinetics of the reactions in the vicinity of a membrane domains are likely to be different from elsewhere, possibly due to the structuring of water (Wichmann et al., 2003).

Transertion may help explain the controversial existence of many spiral hyperstructures (Errington, 2015). Suppose a particular protein has a tendency to form a spiral via protein–protein and protein–lipid interactions. In the absence of transertion, this tendency may result in a spiral whereas, in the presence of transertion, the high concentration of nascent proteins and associated lipids in a circular membrane domain may compete effectively for the mature proteins and thereby prevent a spiral forming. Alternatively, transertion might help generate a protein spiral if the ensemble of gene, nascent RNA (and perhaps mature RNA) were constrained to be essentially linear via RNA–RNA interactions etc. Given the potential of a transertion hyperstructure for altering the morphology of the membrane or for directing a protein to the wrong location, avoidance of these inappropriate results of transertion may act as a powerful factor in evolution that affects both the position in a protein of membrane-interacting sequences and the type of translocon with which the nascent protein interacts.

Finally, several coupled processes in addition to transertion exist in modern bacteria. These include transcription-translation-assembly as in the case of ribosome synthesis (Woldringh and Nanninga, 1985; French and Miller, 1989; Norris et al., 2007a; Cabrera et al., 2009) and transcription-replication as in the case of initiation of DNA replication and DNA repair (Kogoma, 1997; McGlynn, 2010). It might therefore be expected that these processes in their coupled forms would have played a role in the origins of the first cells. This has been suggested for transertion based on its force-generating properties, which are responsible for expanding the nucleoid in modern bacteria (Woldringh et al., 1995; Cabrera et al., 2009; Libby et al., 2012; Bakshi et al., 2014): in this scenario, transertion would have helped maintain membrane integrity in early cells and have been of selective value even without the advantages of coding (Norris et al., 1999).

## Conflict of Interest Statement

The Associate Editor Arieh Zaritsky declares that, despite being affiliated with the same institution as author Itzhak Fishov, the review process was handled objectively and no conflict of interest exists. The authors declare that the research was conducted in the absence of any commercial or financial relationships that could be construed as a potential conflict of interest.
